# Climate effects on nesting phenology in Nebraska turtles

**DOI:** 10.1002/ece3.7105

**Published:** 2021-01-08

**Authors:** Ashley R. Hedrick, Daniel U. Greene, Erin L. Lewis, Andrew S. Hood, John B. Iverson

**Affiliations:** ^1^ Department of Biology Earlham College Richmond IN USA; ^2^ Department of Ecology, Evolution, and Organismal Biology Iowa State University Ames IA USA; ^3^ Environmental Research South Weyerhaeuser Company Columbus MS USA; ^4^ Department of Biology Utah State University Logan UT USA

**Keywords:** *Chelydra*, *Chrysemys*, climate change, nesting phenology, precipitation, weather

## Abstract

A frequent response of organisms to climate change is altering the timing of reproduction, and advancement of reproductive timing has been a common reaction to warming temperatures in temperate regions. We tested whether this pattern applied to two common North American turtle species over the past three decades in Nebraska, USA. The timing of nesting (either first date or average date) of the Common Snapping Turtle (*Chelydra serpentina*) was negatively correlated with mean December maximum temperatures of the preceding year and mean May minimum and maximum temperatures in the nesting year and positively correlated with precipitation in July of the previous year. Increased temperatures during the late winter and spring likely permit earlier emergence from hibernation, increased metabolic rates and feeding opportunities, and accelerated vitellogenesis, ovulation, and egg shelling, all of which could drive earlier nesting. However, for the Painted Turtle (*Chrysemys picta*), the timing of nesting was positively correlated with mean minimum temperatures in September, October, December of the previous year, February of the nesting year, and April precipitation. These results suggest warmer fall, and winter temperature may impose an increased metabolic cost to painted turtles that impedes fall vitellogenesis, and April rains may slow the completion of vitellogenesis through decreased basking opportunities. For both species, nest deposition was highly correlated with body size, and larger females nested earlier in the season. Although average annual ambient temperatures have increased over the last four decades of our overall fieldwork at our study site, spring temperatures have not yet increased, and hence, nesting phenology has not advanced at our site for *Chelydra*. While *Chrysemys* exhibited a weak trend toward later nesting, this response was likely due to increased recruitment of smaller females into the population due to nest protection and predator control (*Procyon lotor*) in the early 2000s. Should climate change result in an increase in spring temperatures, nesting phenology would presumably respond accordingly, conditional on body size variation within these populations.

## Graphical abstract

A frequent response of organisms to climate change is altering the timing of reproduction. Over the past three decades, the timing of nesting of the Common Snapping Turtle (*Chelydra serpentina*) was negatively correlated with mean December maximum temperatures of the preceding year and mean May minimum and maximum temperatures in the nesting year and positively correlated with precipitation in July of the previous year. For the Painted Turtle (*Chrysemys picta*), the timing of nesting was positively correlated with mean minimum temperatures in September, October, December of the previous year, February of the nesting year, and April precipitation.

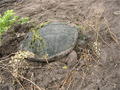

## 1 INTRODUCTION

There is near consensus in the scientific community that climate change is a reality and is accelerating, and its biological effects are already being felt (Pachauri et al., 2014). Considerable evidence already exists demonstrating the impacts of climate change (especially warming) on the reproductive phenology of plants (Ahas, 1999; Price & Waser, 1998; Sherry et al., 2007), invertebrates (Roy & Sparks, 2000), amphibians (Benard, 2015; Gibbs & Breisch, 2001; While & Uller, 2014), fish (Parmesan & Yohe, 2003; Root et al., 2003), birds (Bowers et al., 2016; Bradey et al., 1999; Charmantier & Gienapp, 2014; Crick et al., 1997; Fritts et al., 2018), mammals (Réale et al., 2003), lizards (Bull & Burzacott, 2002; Telemeco et al., 2009), snakes (Morenro‐Rueda et al., 2009), and turtles (citations in Table 1). The emerging pattern in temperate regions is that as temperatures have warmed, reproductive phenologies (e.g., courtship, nesting, and birth) have advanced in time (i.e*.,* occur earlier in the spring or summer).

**TABLE 1:** Previously published studies demonstrating correlations between preseason climate and nest phenology in turtles

Species	Nest Trait	Phenology inversely correlated with temperatures prior to nesting	Earlier nesting phenology through time	Reference
*Caretta caretta* (FL)	Median Nest Date	Yes	Yes	Weishampel et al. (2004)
*Caretta caretta* (FL)	Median Nest Date	Yes	Yes	Pike et al. (2006)
*Caretta caretta* (FL)	First Day Nesting	Yes	No	Pike et al. (2006)
*Caretta caretta* (NC)	First Day Nesting	Yes	No	Hawkes et al. (2007)
*Caretta caretta* (Greece)	First Day Nesting	Yes	Yes	Mazaris et al. (2008), Patel et al. (2016)
*Caretta caretta* (FL)	First Day Nesting	Yes	NA	Pike (2008)
*Caretta caretta* (Greece)	First Day Nesting	Yes	NA	Mazaris et al. (2009)
*Caretta caretta* (FL)	Median Nest Date	Yes	No	Weishampel et al. (2010)
*Caretta caretta* (multiple)	First Day Nesting	Yes	NA	Mazaris et al. (2012)
*Caretta caretta* (FL)	First Day Nesting	Yes	NA	Lamont and Fujisaki (2014)
*Caretta caretta* (FL)	Median Nest Date	No	NA	Lamont and Fujisaki (2014)
*Chelonia mydas* (FL)	First Day Nesting/Median Nest Day	No	NA	Pike (2009)
*Chelonia mydas* (FL)	Median Nest Date	Yes	No	Weishampel et al. (2010)
*Chelonia mydas* (East Africa)	Peak Nest Date	Opposite	No	Dalleau et al. (2012)
*Chelydra serpentina* (ON)	First Day Nesting	Yes	No	Obbard and Brooks (1987), Janzen et al. (2018), Edge et al. (2017)
*Chelydra serpentina* (MI)	First Day Nesting	Yes	NA	Congdon et al. (1987), Edge et al. (2017)
*Chelydra serpentina* (NE)	First Day Nesting/Mean Nesting Day	Yes	No	Janzen et al. (2018), this paper
*Chelydra serpentina* (IL)	First Day Nesting	Yes	Yes	Janzen et al. (2018)
*Chelydra serpentina* (SC)	First Day Nesting	No	No	Janzen et al. (2018)
*Chrysemys picta* (ON)	First Day Nesting	Yes	No	Christens and Bider (1987), Janzen et al. (2018), Edge et al. (2017)
*Chrysemys picta* (MI)	First Day Nesting/Median Nest Date	Yes	NA	Rowe et al. (2003), Edge et al. (2017)
*Chrysemys picta* (NE)	First Day Nesting	Yes	No	Iverson and Smith (1993), this paper
*Chrysemys picta* (IL)	Median Nest Date	Yes	Yes	Schwanz and Janzen (2008), Janzen et al. (2018)
*Chrysemys picta* (IL; 2 Rivers)	First Day Nesting	Yes	No	Janzen et al. (2018)
*Dermochelys coriacea* (multiple)	First 10th Percentile Nesting	Opposite	NA	Neeman et al. (2015)
*Emydoidea blandingii* (MI)	First Day Nesting	Yes	NA	Congdon et al. (1983)
*Emydoidea blandingii* (IL/WI)	90% Nested	Yes	NA	Buckardt et al. (2020)
*Gopherus agassizii* (IL)	Mean Gravid Date	Yes	NA	Lovich et al. (2012)
*Gopherus polyphemus* (GA)	First Day Nesting	No	NA	Levengood et al. (2015)
*Graptemys geographica* (PA)	First Day Nesting	Yes	NA	Nagle and Congdon (2016)
*Graptemys ouachitensis* (WI)	First Day Nesting	Yes	NA	Geller (2012)
*Malaclemys terrapin* (NJ)	First Day Nesting	Yes	Yes	Wood et al. (2013)
*Malaclemys terrapin* (MD)	First Day Nesting	Yes	No	Janzen et al. (2018)
*Trachemys scripta* (IL)	First Day Nesting	NE	Yes	Tucker et al. (2008), Janzen et al. (2018)
*Trachemys scripta* (SC)	First Day Nesting	No	No	Janzen et al. (2018)
*Kinosternon flavescens* (NE)	First Day Nesting	No	No	Janzen et al. (2018)
*Kinosternon subrubrum* (SC)	First Day Nesting	No	No	Janzen et al. (2018)
*Sternotherus odoratus* (IL)	First Day Nesting	No	No	Janzen et al. (2018)

Note

Abbreviations below species names indicate study site.

Abbreviation: NA, not assessed.

Earlier reproduction has the potential to severely disrupt an organism's life cycle. On the positive side, for temperate animals it might permit the production of additional clutches or broods, and neonates may have more time to feed and grow in the fall prior to their first winter (Carroll & Ultsch, 2007; Rhen & Lang, 1999; Schwanz & Janzen, 2008; Tucker et al., 2008). However, on the negative side, early reproduction might expose eggs or neonates to atypical or mismatched conditions, increasing mortality rates (Benard, 2015; Jara et al., 2019; Muir et al., 2012; Pike et al., 2006; Saino et al., 2011). The potential impact of earlier nesting is especially complicated for species that exhibit temperature‐dependent sex determination during development in the nest, as most turtles do (Janzen & Paukstis, 1991). Hence, understanding the impacts of climate on the reproductive phenology of turtles (and other organisms) is critical to conservation and management in the face of climate change, but also because turtles are among the most endangered organisms on the planet (Stanford et al., 2020).

We have been studying the reproduction and demography of turtle populations in western Nebraska since 1981 (Iverson, 1991; Iverson et al., 1997; Iverson & Smith, 1993). For this study, we sought to examine the effects of climate variables on the nesting phenology in two species: Common Snapping Turtle (*Chelydra serpentina*) and Painted Turtle (*Chrysemys picta*). Detailed descriptions of the reproductive biology of these two species at this site have been previously reported (Iverson et al., 1997; Iverson & Smith, 1993). Both species nest annually in late May to late June or early July, but the timing of nesting varies among years by as much as two weeks in *Chelydra* and over a month in *Chrysemys* (see below). That variation is likely related to variability in weather, but the specific climatic variables that drive nest timing, and how those variables might be changing over time, have not been studied at this site.

Preliminary data from our site suggested that cooler springs delayed the onset of nesting in turtles (date of first nest only; Janzen et al., 2018). For this expanded study, we predicted that spring temperatures would be inversely correlated with the Julian date of the first nest produced each year and the mean date of that first clutch. We also explored the potential effects of monthly precipitation and mean monthly maximum and minimum temperatures during the previous summer, autumn, and winter, when females are undergoing vitellogenesis of the clutch produced the following year (Rollinson et al., 2012). We hypothesized that warmer autumn conditions might contribute to more complete follicle development before winter, and hence, the production of earlier clutches the next spring. In addition, warmer conditions in winter (e.g., Mitchell et al., 2017) or spring (Edge et al., 2017; Janzen et al., 2018) were expected to advance nesting phenology during the following season. We also investigated whether body size, clutch size, or egg size affected nest timing, speculating that larger turtles or those with relatively large eggs or clutches might nest earlier in the season.

Finally, given the deep continental location of our study site, and the finding that climate change is generally progressing more rapidly in continental versus coastal North America (Loarie et al., 2009), we expected turtle nesting at our site to have advanced in time over the course of our long‐term study. Furthermore, that advancement should be more evident than for populations farther east.

## 2 METHODS

### 2.1 Data collection

We monitored nesting turtles that emerged from Gimlet Lake (41°45.24′N, 102°26.12′W), a shallow, sandhill lake on the Crescent Lake National Wildlife Refuge, Garden County, Nebraska, USA (see Iverson & Smith, 1993 for study site description) during 18 (*Chelydra*; Table 2) or 23 years (*Chrysemys*; Table 3). The primary nesting areas were monitored daily during the nesting season (May–July) from at least 06:00 to 22:00 hr by two to five observers. Turtles were weighed, measured (maximum carapace length and maximum plastron length), and marked after nesting. It was not possible to monitor both species for the entire nesting season for every year between 1986 and 2017, but data were available for most years (Tables 2 and 3). In some years, we also sampled *Chelydra* that nested at nearby Island Lake (41°43.95′N, 102°24.16′W).

**TABLE 2:** First, last, and mean (±1 *SD*) nesting dates for *Chelydra serpentina* at Crescent Lake National Wildlife Refuge, Garden County, Nebraska by year

Year	*N*	Mean ± *SD*	First	Last
1993	36	170.8 ± 4.5	163	179
1994	32	156.9 ± 3.0	152	164
1998	14	161.4 ± 4.4	157	174
1999	29	165.4 ± 5.0	155	173
2000	21	165.1 ± 8.3	151	163
2004	15	158.5 ± 2.8	150	162
2005	36	164.5 ± 3.4	158	170
2006	29	155.0 ± 2.7	152	162
2007	91	160.0 ± 7.1	148	165
2008	54	171.6 ± 2.3	167	177
2009	65	168.9 ± 2.6	162	175
2010	52	170.0 ± 4.0	161	178
2011	21	170.6 ± 3.5	165	176
2012	57	156.4 ± 2.3	153	161
2013	49	163.2 ± 2.9	158	168
2014	29	163.4 ± 5.1	154	174
2015	45	162.2 ± 3.0	158	176
2017	25	160.4 ± 3.9	156	171

Note

Dates are Julian days (152 = 1 June, except 153 in leap years). Overall means (and number of sample years) at bottom.

**TABLE 3:** First, last, and mean (±1 *SD*) nesting dates for *Chrysemys picta* at Crescent Lake National Wildlife Refuge, Garden County, Nebraska by year

Year	*N*	Mean ± *SD*	First	Last
1986	26	155.0 ± 5.2	141	164
1988	29	151.5 ± 4.2	144	160
1990	40	156.5 ± 3.6	150	163
1993	20	154.0 ± 4.9	142	161
1994*	4+	NA	137	NA
1998*	4+	NA	140	NA
1999*	5+	NA	148	NA
2000	31	153.5 ± 5.4	143	161
2001	39	152.5 ± 4.3	145	159
2002	31	155.7 ± 4.2	148	163
2003	24	159.2 ± 5.6	149	165
2004	31	164.2 ± 3.2	157	167
2005	48	177.1 ± 3.3	171	185
2006	36	159.3 ± 3.1	151	166
2007	56	157.8 ± 5.1	147	169
2008	67	172.2 ± 3.8	161	178
2009	59	168.4 ± 4.0	157	175
2010	52	158.9 ± 4.1	152	167
2012	71	146.1 ± 8.4	128	156
2013	98	159.8 ± 3.7	153	167
2014	82	158.5 ± 4.7	147	166
2015	98	167.0 ± 4.6	153	174
2017	43	157.7 ± 3.7	151	165
Means	159.2 (20)	148.5 (23)	166.6 (20)	

Note

Dates are Julian days (152 = 1 June, except 153 in leap years). Asterisks indicate years when census ended before all first clutches laid. Overall means (and number of sample years) at bottom.

For each species, we recorded the date in May or June each year that the first gravid female of each species emerged from the lake with the intention of nesting (i.e., gravid and attempted or completed a nest). Additionally, we calculated the mean nest date each year for all emergence dates (even if a nest was not completed that day). For females that failed to complete a nest when first sighted in a given year (e.g., if she was disturbed by Refuge personnel activities) and then nested on a subsequent night during the following several days (i.e., before she could produce a second clutch), her nest was scored as having been deposited on the night she was first observed constructing a nest.

*Chelydra* produced a maximum of one clutch per year at this site (Iverson et al., 1997), but some female *Chrysemys* produced at least three clutches per season (Iverson & Smith, 1993). Hence, for the latter species, mean nest date refers only to the first clutch of the season. The end of production of first clutches for *Chrysemys* was estimated by assuming that at least ten days are required to produce a second clutch by a given female (though usually 12 or more days; Iverson & Smith, 1993) and noting the dates for females known to be depositing their second clutches. The daily frequency of nesting females in the interval between 11 days after the first nest date and the date of the first known second clutch was examined for a gap (or at least a greatly reduced nesting frequency) that was presumed to indicate the transition between first and second clutches. The last day for a first clutch was estimated to be the last day before that gap. We realize that this method in imprecise and likely excludes some females that produced their first clutches, while most of the population was producing second clutches, but our sample sizes should be large enough to minimize this potential bias.

Climate data from July of the previous year through May of the year of nesting were obtained from the NOAA weather station located immediately adjacent to the turtle nesting area (<100 m). We initially compiled a series of climatic variables for the years 1970 through 2017, including mean monthly maximum and minimum daily temperatures, and monthly precipitation. We excluded climate data for the month of June since it overlapped with the nesting season of our study species.

### 2.2 Statistical approach

We investigated whether nest deposition (first nests per season, mean nests, and nest dates by individuals) for *Chelydra* and *Chrysemys* was influenced by climatic predictor variables and whether they changed over time during our study period. Additionally, we assessed whether population‐level measurements of body size and reproductive variables (carapace length, plastron length, female mass postnesting, mean egg mass, and clutch size) have changed during our study, as these variables can influence timing of nest deposition of an individual.

We analyzed our data two ways. First, we conducted least squares regression analyses to assess long‐term trends in climate variables versus year and relationships between temperature and precipitation variables and time (Julian day of nest deposition and years) for the first nest of a season and mean nest dates. Significant p‐values for regressions were conservatively adjusted for multiple comparisons by using a sequential Bonferroni correction to α (Holm, 1979). We conducted these analyses using Statview software (Abacus Concepts). Second, we assessed relationships between climatic variables and the above‐mentioned life history traits on nesting phenology (Julian day of nest deposition) of all nests. For these analyses, we fit linear mixed‐effect models via maximum likelihood in R using the package “lme4” (Bates et al., 2015; R Core Team, 2017). We evaluated candidate models using the Akaike information criterion (AIC) where the level of importance was assessed by model weights (*w*) and overall ranking in the candidate set. For both species, we included carapace length as a fixed effect to account for an individual's growth and size over time. For *Chrysemys*, we used the random effects of Female ID (identification) to account for individual variation and year to account for differences in sample sizes. And for *Chelydra*, we used year as the random effect, but not female ID because 76% of nests could not be associated with a female ID (see below).

To improve model convergence and determine relationships with nest foray dates, we z‐standardized the continuous covariates for our mixed‐effect model analyses. We examined relationships between our covariates and dropped one of two variables if their Pearson's correlation coefficient was >|.70|, with one exception; mean minimum and maximum air temperatures are correlated, but serve important, separate roles in regulating water temperatures and metabolic rates of freshwater turtles (however, minimum and maximum means for a given month were not included in the same models because of collinearity). In total, we had 33 climatic variables for our analyses.

Each weather variable can affect different aspects of reproductive ecology, including timing of emergence from hibernation, food resources, water temperatures, metabolism, basking conditions, and egg development, including vitellogenesis, ovulation, and egg shelling. Therefore, we did not develop multiple regression models (additive or interactive) to assess which combination of variables in a model was “best” at predicting nesting phenology. Instead, we sought to evaluate how each climatic variable influenced timing of nesting, including how a variable ranked in importance among all candidate variables, and the magnitude of its effect. We determined the covariate's predictive importance by inspecting conditional beta coefficient (*β*) estimates and their 95% confidence intervals (CI), with significance defined as CIs for a variable that did not overlap zero. We evaluated predicted values of our significant variables to assess relationships with Julian day of nesting using the “ggeffects” package in R (Lüdecke, 2018).

## 3 RESULTS

### 3.1 Nesting summary

We tallied 705 nesting forays for *Chelydra* and individually identified 230 (23.6%) females, although many of the others were marked but eluded us after nesting by returning to the water before capture. Of the known females, 45 were only recorded on a nesting foray once during the study, but the average number of years that known females emerged to nest was 2.3 (range 1–8). We found no differences in nesting dates, changes in nesting dates over time (years), or body size metrics between Gimlet and Island Lakes, except for clutch size, our top model, which was significantly smaller at Island Lake (Tables S1–S4). However, sample sizes were disproportionate, with 204 nests from Gimlet Lake and 58 from Island Lake, and likely influenced this result. Therefore, we merged datasets of both lakes for climatic variable analyses.

We also recorded 981 total forays for first nests for *Chrysemys* and associated 503 of those to known females (51.3%). Of the known females, 160 were identified on a first clutch nesting foray only once during the study, but the average number of years that identified females emerged on forays for first nests was 1.9 (range 1–10). As the study progressed, clutch size for *Chrysemys* remained unchanged, but there was a significant increase in carapace length of nesting females (Tables S3 and S4).

**FIGURE 1:**
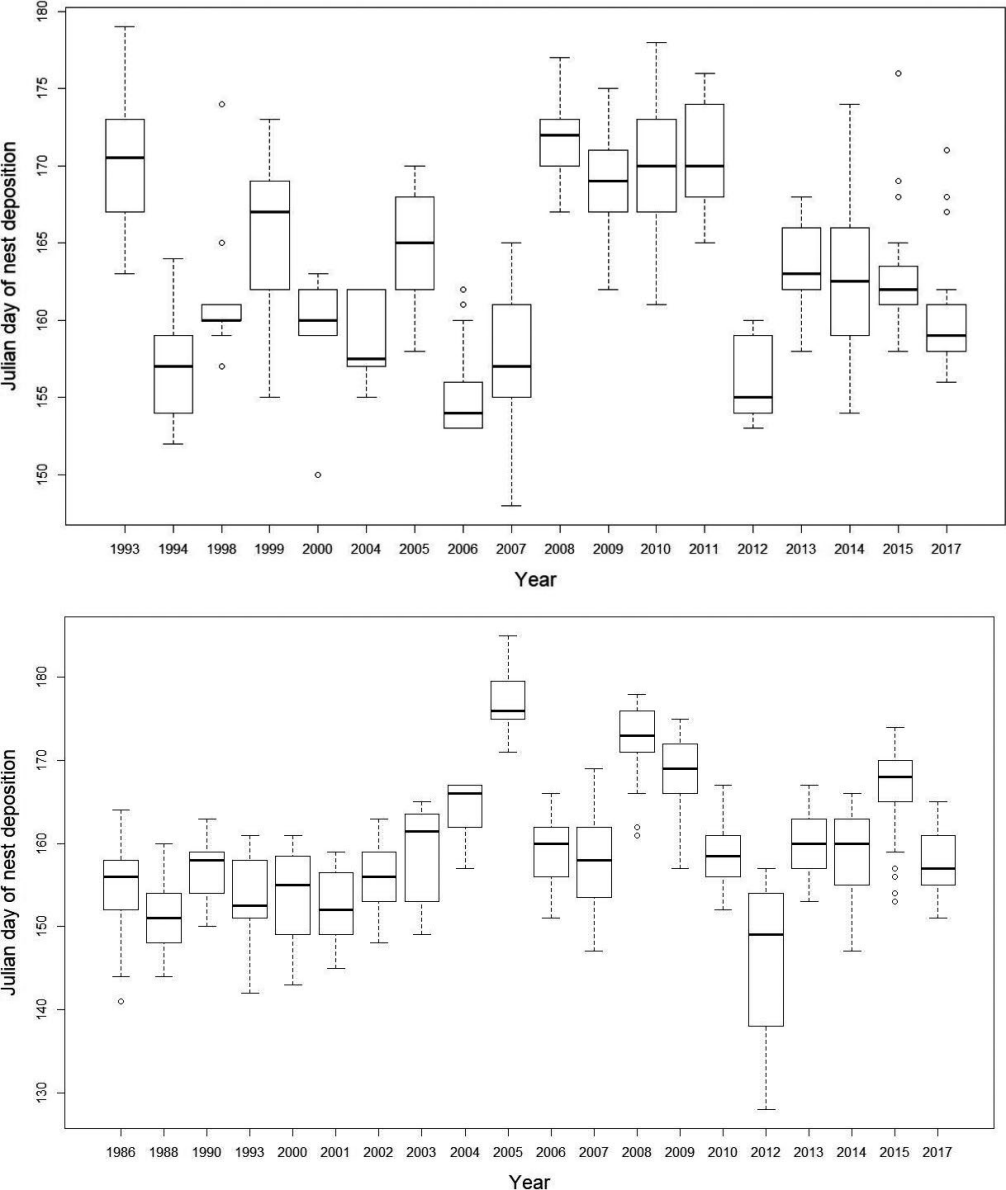
Box‐and‐whisker plots of Julian day of nest deposition of *Chelydra serpentina* (top; mean of annual means is Julian day 164; *N* = 18) and *Chrysemys picta* (bottom; mean of annual means is Julian day 159; *N* = 20) by year at Crescent Lake National Wildlife Refuge, Garden County, Nebraska

Julian day of nest deposition was highly variable among years (e.g., Figure 1). The earliest nesting date for *Chelydra* occurred on Julian day 148 (28 May 2007) and the latest first nest was deposited on Julian day 179 (28 June 1993) (Table 2). The earliest nesting date for *Chrysemys* occurred on Julian day 128 (7 May 2012) and the latest first nest was deposited on Julian day 185 (4 July 2005). The average date of the first nest for *Chelydra* across 18 years was Julian day 157 (6 June), and the average date of nest deposition across those 18 years was Julian day 164 (13 June; Table 2). For *Chrysemys*, the average date of the first nest was Julian day 149 (29 May), and the average nest date was Julian day 159 (8 June; Table 3). Date of the first nest and mean date of nesting within years were highly correlated (*p* < .0001) for both *Chelydra* (*R* = .82) and *Chrysemys* (*R* = .93), although the first nesting dates in a given year between these two species were not correlated (*N* = 17; *R* = 0.38; *p* = 0.14) nor were the mean dates (*N* = 14; *R* = 0.34; *p* = 0.24).

### 3.2 Climate summary

**FIGURE 2:**
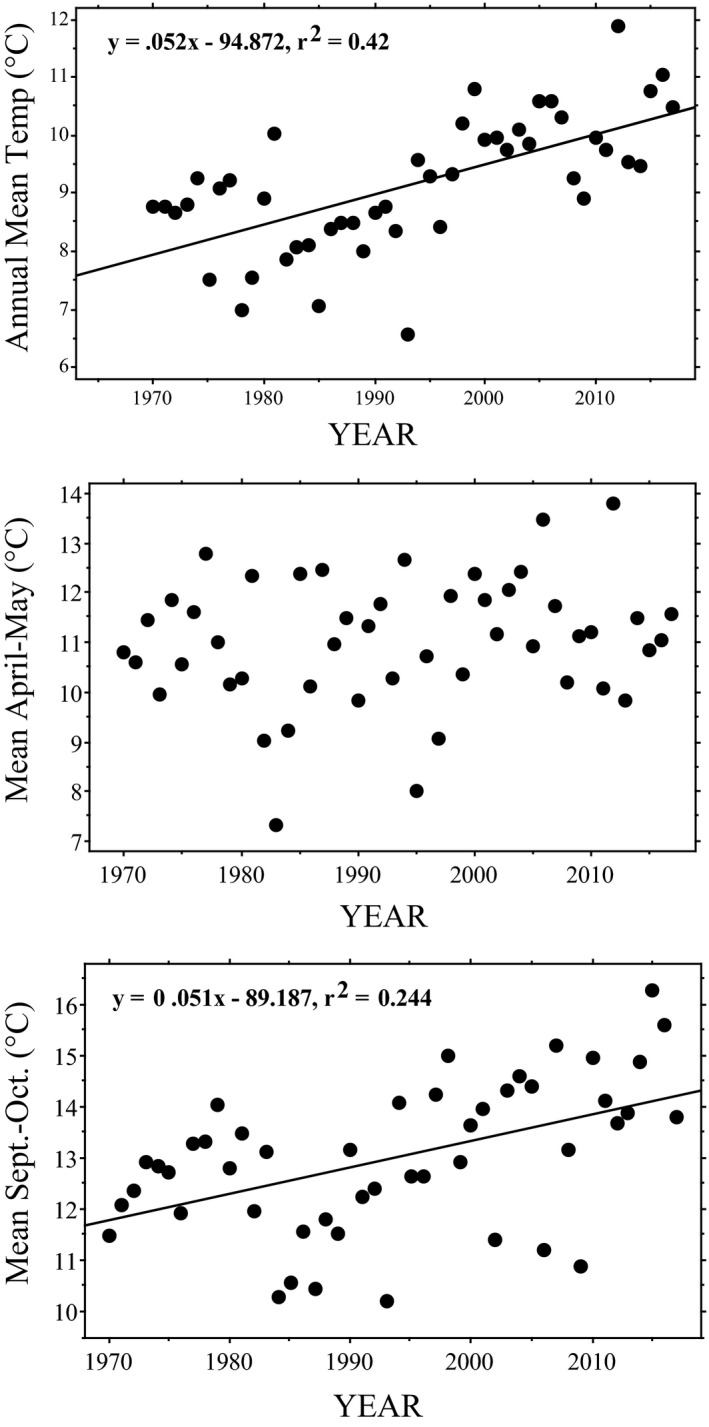
Variation in environmental temperatures from 1970 to 2017 at the Crescent Lake National Wildlife Refuge, Garden County, Nebraska. TOP: Increase in mean annual temperatures (°C) (least squares regression: *R* = 0.65; *p* < .0001). For mean April–September temperature: *y* = 0.042*x*–67.16; *R* = 0.60; *p* < .0001. MIDDLE: Variation in spring temperature (mean April–May temperature in °C), indicating no pattern of change over time (least squares regression, *R* = 0.171; *p* = 0.24). BOTTOM: Variation in autumn temperature (mean September–October temperature in °C), indicating a significant warming over time (least squares regression, *R* = 0.494; *p* = 0.0004)

Annual precipitation at this site averaged 43.3 cm between 1970 and 2017, and wet season (May–June) rainfall averaged 14.9 cm. However, no measure of precipitation (monthly, seasonal, or annual) changed significantly with time over those 48 years (*p* > .17 for all regressions). In contrast, mean annual temperature at our study site has warmed at a rate of about 0.5°C per decade (Figure 2). Mean daily minimum temperatures for every month of the year except February and December increased significantly from 1970 to 2017 (Table 4). However, mean daily maximum temperatures increased significantly only for January (*p* = .037), but only if no adjustment in that p‐value was made for multiple comparisons (Table 4). Mean April–May temperature also did not change over that period (Figure 2), although mean September–October temperature increased significantly, by about 0.5°C per decade (Figure 2).

**TABLE 4:** Correlations between year (1970–2017; *N* = 48) and mean monthly minimum and maximum temperatures at Crescent Lake National Wildlife Refuge, Garden County, Nebraska

Month	Minimum		Maximum	
	*R*	*p*	*R*	*p*
Jan	.626	<.0001	.305	.037
Feb	.322	.025	.220	.132
Mar	.574	<.0001	.179	.225
Apr	.501	<.0001	.179	.224
May	.522	<.0001	.210	.152
Jun	.601	<.0001	.080	.588
Jul	.727	<.0001	.153	.300
Aug	.675	<.0001	.036	.806
Sep	.720	<.0001	.049	.740
Oct	.648	<.0001	.231	.115
Nov	.594	<.0001	.177	.230
Dec	.393	.006	.095	.519

Note

Regression coefficients (*R*) are followed by *p*‐values. Only *p*‐values < .0001 are significant after sequential Bonferroni adjustment for multiple comparisons.

### 3.3 Climate effects

**FIGURE 3:**
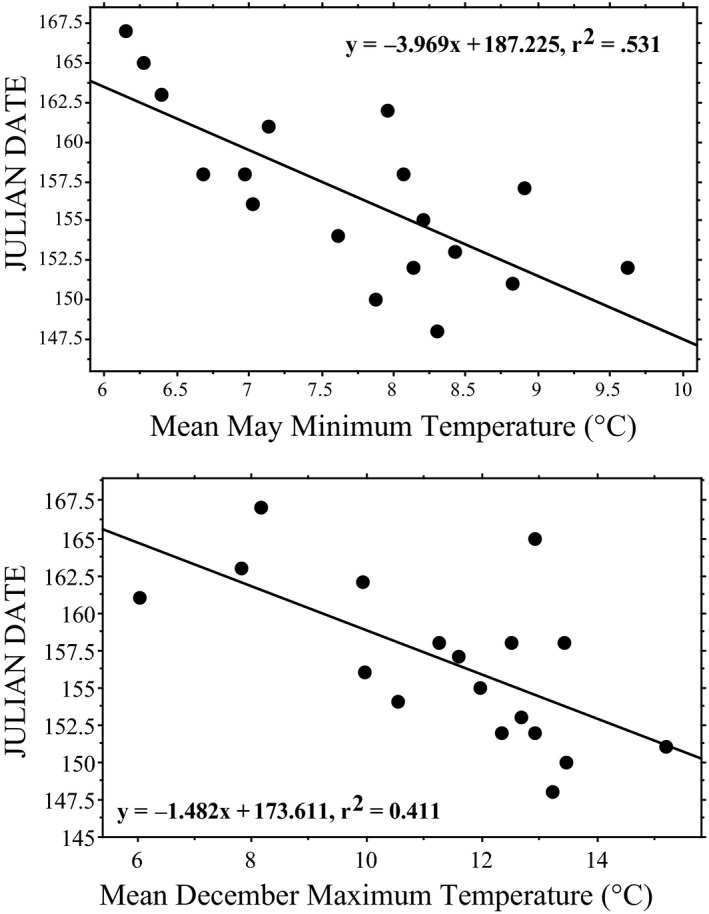
First Julian nesting date of *Chelydra serpentina* from 1993 to 2017 at Crescent Lake National Wildlife Refuge, Garden County, Nebraska, in response to mean May minimum daily temperature (TOP) and mean December maximum daily temperature (BOTTOM). For May temperatures, regression is statistically significant before and after sequential Bonferroni adjustment (*p* = .0006; see Table 4); for mean Julian nesting date *y* = −1.899*x* + 250.642, *R* ^2^ = .412, and *p* = .004. For December temperatures, regression is statistically significant before but not after sequential Bonferroni adjustment (*p* = .0041; see Table 4); for mean Julian nesting date *y* = −1.260*x* + 177.987, *R* ^2^ = .311, and *p* = .0162

Based on our mixed model analysis, variation in the nesting date by year for *Chelydra* was best explained by mean May minimum temperatures (Table 5; Tables S5 and S6), where each degree C increase in mean May minimum temperature advanced the first nesting date by ca. four days, and the mean nest date by ca. two days (Figure 3). In addition, each degree increase in mean maximum December temperature advanced first nesting by 1.5 days and mean nesting date by 1.3 days. Together, these two variables explained 62% of the variation in first nesting date (Annual First Nesting Date = −0.810*Decmax – 2.942*Maymin + 188.585; *p* = .0007) and 48% of the variation in mean nesting date (Annual Mean Nesting Date = −0.676*Decmax – 2.56*Maymin + 191.019; *p* = .0079).

**TABLE 5:** Spring climate variables correlated with nest date for two turtle species at Crescent Lake National Wildlife Refuge, Garden County, Nebraska

*Chelydra*		*Chrysemys*	
First nest	Mean nest	First nest	Mean nest
May min	May min	**September min**	**September min**
.0006*	.004	**.05**	.03
May max	May max	May max	**October min**
.004	.005	.05	.05
December max	**July rain**		
.004	**.01**		
December min	December max		
.06	.02		
	April max		
	.03		
	April min		
	.05		
	March max		
	.05		
	January max		
	.05		

Note

*p*‐Values appear below variable name, and all listed climate variables are means. Positive correlations are bolded; all others are inverse relationships. *p*‐Values with asterisks are significant after sequential Bonferroni adjustment for multiple comparisons (separately for temperature and precipitation). Sample sizes and sample years in Table 1.

For all *Chelydra* nests (mixed model analyses), eight of our climatic variables significantly influenced nest deposition (Tables S5 and S6). Notably, an increase in mean May minimum (6.1 to 9.6°C) and mean May maximum temperatures (16.9 to 24.0°C) were the most influential variables, each advancing nesting from approximately Julian days 169 to 155 and day 170 to 158, respectively. Similarly, both an increase in mean December maximum (−1.7 to 7.4°C) and mean April maximum (11.1 to 18.8°C) temperatures advanced nesting from Julian day 171 to 160 and day 170 to 158, respectively. Conversely, an increase in precipitation during July of the preceding year delayed nesting from Julian day 159 to 170 when precipitation increased from 0.52 to 5.77 cm.

**FIGURE 4:**
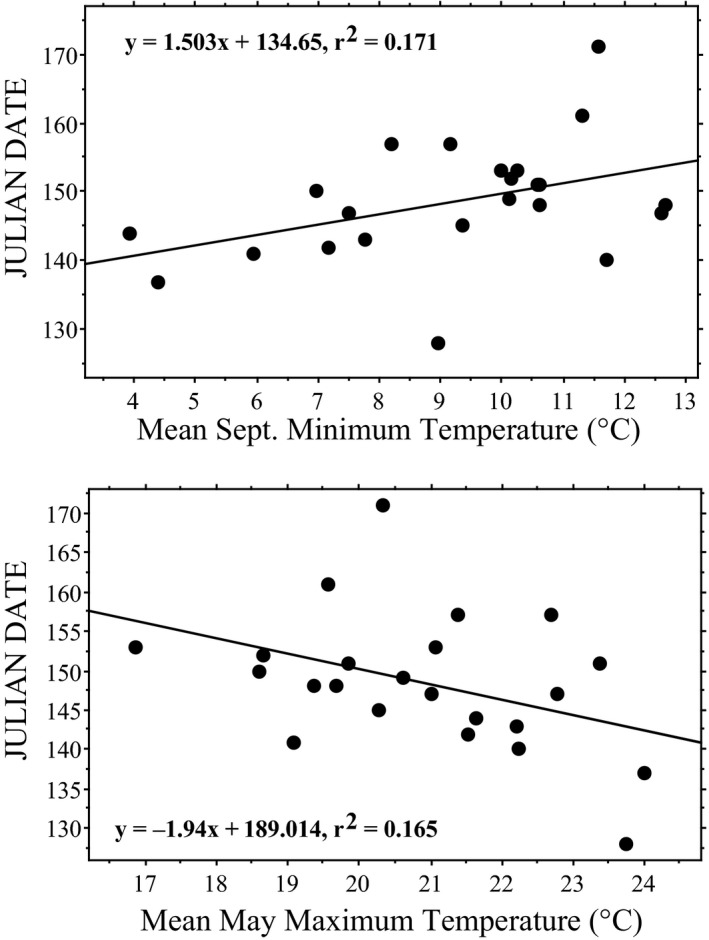
First Julian nesting date of *Chrysemys picta* from 1986 to 2017 at Crescent Lake National Wildlife Refuge, Garden County, Nebraska, in response to mean September minimum temperature (TOP: *p* = .049) and mean May maximum temperature (BOTTOM: *p* = .055). Regressions are not statistically significant after sequential Bonferroni adjustment (Table 5). For mean Julian nesting date *y* = 1.740*x* + 143.330, *R* ^2^ = .25, and *p* = .026, and *y* = −1.315*x* + 186.523, *R* ^2^ = .10, and *p* = .18, respectively

Based on our mixed model analysis, for *Chrysemys*, nesting date by year was best explained by mean September minimum temperatures (Table 5), where each degree increase in mean temperature delayed first nesting date by 1.5 days and mean nesting date by 1.3 days. Furthermore, each degree increase in mean May maximum temperature advanced first nesting by 1.9 days, but did not significantly affect mean nesting date (Figure 4). Together, these two variables explained 51% of the annual variation in first nesting (Annual First Nesting Date = 01.167*Septmin – 1.482*Maymax + 168.712; *p* = .05) and 55% of the variation in mean nesting (Annual mean Nesting Date = 1.601*Septmin – 0.99*Maymax + 165.136; *p* = .04).

For all *Chrysemys* nests (mixed model analyses), five of our climatic variables significantly influenced nest deposition (Tables S7 and S8). An increase in either mean February minimum temperature from −15.2 to −3.9°C or mean minimum temperatures in the previous September (from 3.9 to 12.6°C) delayed nesting up to 15 days from Julian day 150 to 165. Similarly, we found that an increase in either October minimum temperatures (−1.9 to 5.6°C) or mean December minimum temperatures in the preceding year (−17.1 to −4.8°C) each delayed nesting from Julian 153 until day 165. Lastly, April precipitation delayed nesting from Julian day 155 to 165 when rainfall increased from 0.44 to 4.3 cm.

### 3.4 Life history effects

Body size and reproductive variables (carapace length, plastron length, clutch size, and spent mass of a postnesting female) were all significantly inversely correlated with nesting dates (Tables S9 and S10). We dropped spent body mass and plastron length as predictor variables for both species because of high correlation coefficients (>|.87|) with carapace length. For *Chelydra*, females with a carapace length of 225 mm were predicted to nest on Julian day 167, whereas a larger female with a carapace length of 395 mm was predicted to nest on day 162. Similarly, females of *Chrysemys* with a carapace length of 150 mm were predicted to nest on Julian day 162, whereas a larger female with a carapace length of 206 mm was predicted to nest on Julian day 157.

### 3.5 Temporal effects

**FIGURE 5:**
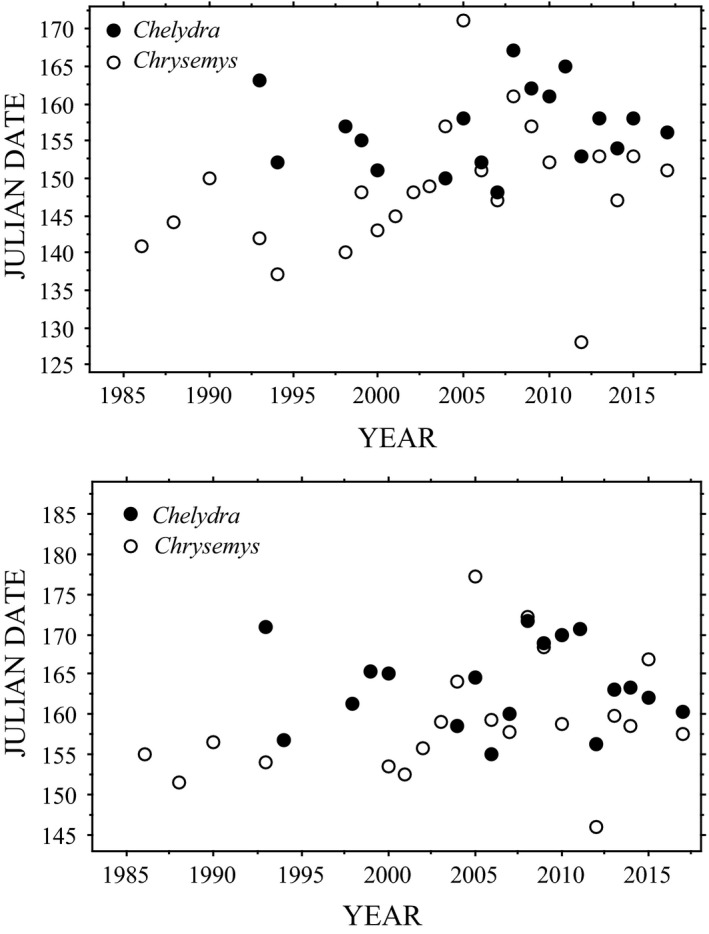
First (TOP) and mean (BOTTOM) Julian nesting dates of *Chelydra serpentina* (solid dots; *N* = 18) and *Chrysemys picta* (open dots; *N* = 23) by year at Crescent Lake National Wildlife Refuge, Garden County, Nebraska. None of the relationships was significant (TOP: *R* = .13, *p* = .60; *R* = .32, *p* = .14, respectively; BOTTOM: *R* = .02, *p* = .93; *R* = .33, *p* = .16, respectively)

Least squares analyses of first and mean nesting dates for both *Chelydra* and *Chrysemys* revealed no change over time during our study period (Figure 5). Similarly, from our mixed model analyses, we found no changes in nesting dates over time for *Chelydra* (*β* = 0.04, *SE* = 0.18, 95% CI = −0.31, 0.40) or *Chrysemys* (*β* = 0.26, *SE* = 0.17, 95% CI = −0.08, 0.61).

**FIGURE 6:**
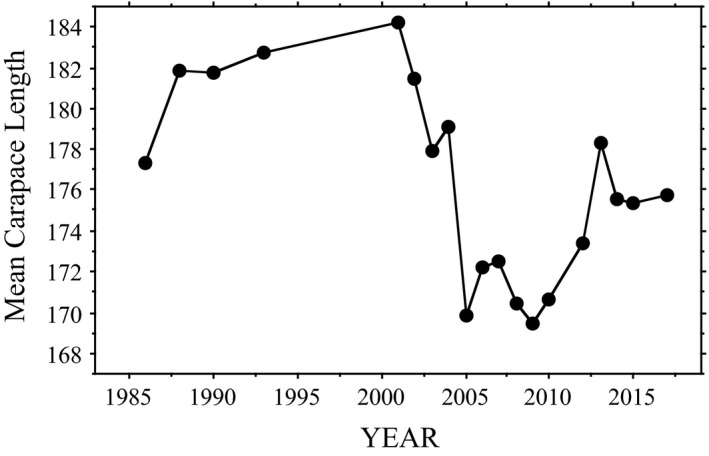
Change in mean carapace length (in mm) over time of nesting female *Chrysemys picta* at Crescent Lake National Wildlife Refuge, Garden County, Nebraska. Linear regression is significant (*R* = −.56; *p* = .0135) but masks the decline in the early 2000s and subsequent ongoing recovery. For *Chelydra serpentina*, linear regression was not significant (*R* = −.46; *p* = .074)

Mean annual carapace length did not change over time for *Chelydra* (*R* = −.46; *p* = .07; *n* = 18), a pattern supported in our mixed model analyses (Tables S3 and S4). However, for *Chrysemys*, mean annual carapace length decreased over time (Figure 6). In contrast, our mixed model analysis suggested that carapace length in *Chrysemys* increased over time (Tables S3 and S4), although the latter results are complicated by the uneven annual sample sizes (Table 3) and the clear trend of an increase in body size over the last third of the study (Figure 6) during the years with large sample sizes (Table 3). Furthermore, the relationship between body size and nest date over the full study period in *Chrysemys* was confounded by population‐level demographic changes due to variation in female mortality and nest survivorship (Figure 6; see discussion for details).

## 4 DISCUSSION

Spring temperatures are generally inversely correlated with nest phenology in turtles (Table 1), with 27 of 38 previous studies (representing 15 species) demonstrating this pattern. Only two studies (both for marine turtles) exhibited the opposite trend (see Table 1), but both of those studies used different measures of nest timing (peak nesting date; first 10th percentile) than all other studies, including ours.

At our site, warmer springs also advanced nesting in *Chelydra*, as did increased mean December maximum temperatures. The mechanisms driving this pattern likely operate through thermoregulation or local food chain productivity (Schwanz & Janzen, 2008). Increased local environmental temperatures during the winter and spring presumably permit earlier emergence from hibernation, increased metabolic rates (e.g., via basking), and accelerated vitellogenesis, ovulation, and egg shelling, all of which would drive earlier nesting (Mitchell et al., 2017; Obbard & Brooks, 1987). Similarly, an increase in local food chain productivity due to increased temperatures could also provide more resources necessary to speed up reproductive demands, although this mechanism is probably secondary to thermoregulatory affects. However, nest timing in *Chrysemys* was not strongly influenced by spring temperatures, as predicted, but rather, delayed by warm temperatures in the fall and winter.

Rollinson et al. (2012) demonstrated that snapping turtles complete vitellogenesis primarily by the end of the previous fall, whereas for *Chrysemys* the process occurs both in the fall and spring (see also Callard et al., 1978). Our data suggest that vitellogenesis in *Chelydra* may in fact continue into December, even though air temperatures are quite cold that month (long‐term mean daily maximum = 3.8°C; minimum, −10.1°C). However, for *Chrysemys*, warm temperatures in early fall might be expected to accelerate vitellogenesis and hence result in early nesting during the following summer (the opposite of our findings). Perhaps the metabolic costs of a warm fall exceed the benefits to vitellogenesis in *Chrysemys*. Part of the difficulty in explaining these novel patterns is our lack of water temperature data, which presumably lag air temperatures. Interpreting differences between the two species is further complicated by the much higher propensity of *Chrysemys* to bask aerially compared to *Chelydra*. Indeed, basking in *Chrysemys* may explain why nest timing in that species was so much less predictable by air temperatures than in *Chelydra*.

We were surprised to find that increased precipitation in July was correlated with a delay in nesting in *Chelydra,* over ten months later. Although previous studies have examined the impact of precipitation on nest timing during the nesting season (see review in Czaja et al., 2018), no study has examined the effects on nest timing of precipitation outside the nesting season. Increased precipitation in July at our site was correlated with colder mean daily July maximum temperatures (*R* = .38; *p* = .007; *N* = 48 years), but average July temperatures were not related to nest timing in *Chelydra*. We can therefore only speculate that increased precipitation in July delays nesting the following year by slowing vitellogenesis, perhaps via its effect on lowering water temperatures. The importance of April precipitation in delaying nesting in *Chrysemys* was surprising. We suspect that high precipitation in April reduces basking opportunities and decreases water temperatures, both of which would be expected to delay nesting in *Chrysemys*, which must complete vitellogenesis in the spring (Rollinson et al., 2012).

Our analyses revealed that larger female *Chelydra* and *Chrysemys* tended to nest earlier in the season than smaller females. Because clutch size and egg size are correlated with body size in both species (Iverson et al., 1997; Iverson & Smith, 1993), early nests included more and bigger eggs. Earlier nesting by larger female turtles has previously been reported for *Graptemys geographica* in Pennsylvania (Nagle & Congdon, 2016). These results suggest that the size class distribution of a population can impact its nesting phenology, complicating phenology comparisons across years, populations, and species.

Although mean body size of nesting female *Chelydra* did not vary over time at our study site (*R* = −.46; *p* = .07), mean annual carapace length of nesting female *Chrysemys* did decrease significantly (Figure 6; Tables S3 and S4). This decrease was most likely the result of nest protection that began in 1993 and a major depredation period in 2003 and 2004 (evident in Figure 6). In conjunction with our overwinter physiology studies (e.g., Costanzo et al., 1995), we began sporadic protection of *Chrysemys* nests in 1993, followed by rigorous protection of every located nest commencing in 1999 and continuing through 2017. This effort flooded the nesting population with small, primiparous females in the mid to late 2000s (e.g., see sample sizes in Table 3). In addition, the local raccoon (*Procyon lotor*) population apparently grew in 2003–2004 (as evidenced by sightings during nest surveys), resulting in major depredation of nesting females during this period. For example, early in the nesting season in June 2004, we witnessed a particularly bold raccoon (later removed by Refuge personnel) take several females as they left the marsh to nest during daylight hours in late afternoon. Two of those females that were recovered (dead) were long‐term recaptures (28 years old, 190 mm carapace length; 21 years old, 185 mm carapace length). These two circumstances resulted in a precipitous decline in mean body size for nesting females between 2000 and 2005, followed by increasing mean body size as the younger females aged. Although predicted nesting date for *Chrysemys* has not significantly changed over time, there may be an emerging trend in later nesting dates (e.g., Figures 1 and 5) that may be due to the growing number of newly maturing females recruited into the population each year.

Because both species in our study demonstrated size‐related variation in nesting dates, future studies of turtle nesting phenology should consider the effects of body size as a complicating factor when making comparisons over time and space, as changes in nesting phenology may actually be related to or compounded by changes in population structure. For example, a population of turtles experiencing a decline in body size over time (e.g., via increased poaching or predation) would be expected to exhibit a pattern of delayed nesting even if the climate had not changed. In the future, failure to account for changes in size class distributions over time in turtle nesting phenology studies may mask or artificially suggest temporal patterns.

Climate change over the last five decades has produced warmer temperatures overall at our site, with the greatest impact being a noticeable increase in nighttime minimum temperatures (Table 4). It was also our subjective impression that nighttime skies grew increasingly hazy over the study period, and although the cause(s) are not yet clear, the increasingly cloudy skies and warmer nights were likely related. Despite the significant overall warming at our site over the past several decades and a clear inverse relationship between spring temperatures and nesting timing, nesting phenology in at least *Chelydra* has not changed between 1993 and 2017 at our site. This is likely at least in part a reflection of the fact that spring temperatures at our site have not changed over that period (Table 5; Figure 3), even though annual temperatures have (Figure 2). The meteorological reasons for this spring difference are not yet evident. In any case, should spring day‐time temperatures eventually warm at our site, the nest phenology of at least these two turtle species in western Nebraska will likely be affected.

Of the 38 studies summarized in Table 1, 23 (nine species) evaluated nesting phenology through time, and surprisingly, only seven of them (five species) documented the expected temporal shift to earlier nesting. However, other studies of each of the latter five species demonstrated no significant shift in phenology. Because climate change has generally been more rapid in continental versus coastal areas of North America (Loarie et al., 2009), we expected continental turtle populations to show more advanced nest dates than those closer to the coast. For example, *Trachemys scripta* in South Carolina exhibited no shift over time, whereas in Illinois it did (Table 1). However, among the four studied populations of *Chelydra*, three (Ontario, Canada, and Nebraska and South Carolina, USA) exhibited no shift in nest timing, while that in Illinois did (Table 1). Similarly, among four populations of *Chrysemys*, (Ontario, Canada, Nebraska and southern Illinois, USA) exhibited no shift, but that in northern Illinois did. Clearly, the observed geographic variation in nest timing in *Chelydra* and *Chrysemys* does not match the predicted continental scenario. Interestingly, the only two populations of these two taxa exhibiting a temporal change are riparian, whereas those with no change are all lentic systems. Whether this is an important factor explaining these patterns will require additional fieldwork, including the collection of water temperature data.

Regretfully, water temperatures were not recorded during our study, since they might be expected to be better predictors of nesting dates (e.g., as sea surface temperatures have been for marine turtles; Table 1). However, even those data would be complicated by differences in habitat use by our study species. In our experience, *Chelydra* seems to occur in shallow (warmer?) water and does limited aerial basking, whereas *Chrysemys* seem to inhabit deeper water and exhibits extensive aerial basking (see also Ernst & Lovich, 2009). Similarly, more detailed analyses of temperatures (water and air) beyond simply monthly means (especially during the spring and fall temperature windows that are most highly correlated with nest timing) might clarify the mechanism for the relationship between temperature and nest timing more precisely (e.g., see Edge et al., 2017; Schwanz & Janzen, 2008).

It is also possible that the inability to detect a change in the nesting phenology at our site, as well as many other sites in Table 1, could be due to the stalling in increasing global mean surface temperatures from the late 1900s through the 2000s known as the “climate change hiatus” (e.g., Kosaka & Xie, 2013). Steady rather than increasing temperatures during that period could explain the lack of statistically significant change in nesting phenology in studies including data collected during that period, although this hiatus is not evident in our climate histories (Figures 2, 3, 4).

Although our study focused on the proximate effects of climate on turtle nesting phenology, its impacts on other areas of life history remain poorly studied (Butler, 2019). For example, climate change is likely to affect the length of the nesting season (e.g., Hedrick et al., 2018; Lamont & Fujisaki, 2014; Pike et al., 2006); internest intervals (Hays et al., 2002); clutch frequency (Mazaris et al., 2012; Tucker et al., 2008); egg and clutch size (Hedrick et al., 2018; Lamont & Fujisaki, 2014; Mazaris et al., 2012); survival of early nests and early nesting females (Mazaris et al., 2009; Schofield et al., 2009); hatching success (Hawkes et al., 2007); posthatching survival (especially in species like *Chrysemys picta* with hatchlings that overwinter terrestrially; Costanzo et al., 1995; Muir et al., 2012); juvenile growth rates (Avery et al., 1993; Gibbons, 1970); and population sex ratios via temperature‐dependent sex determination (Schwanz & Janzen, 2008; Tucker et al., 2008). None of these potential effects have been examined at our site, nor have the fitness costs of earlier nesting.

As previously noted, only 7 of 23 studies that evaluated turtle nesting phenology over time have documented that nest dates have advanced over recent decades. Clearly, the collection of more data is necessary before we can generalize that climate change has altered nesting phenology in nonmarine turtles. Part of the problem is that such studies depend on demanding long‐term studies. For example, of the 38 studies reviewed in Table 1, only three field sites (Ontario, Canada, and Illinois and Nebraska, USA) have recorded nesting histories exceeding 20 years (see also Janzen et al., 2018). Thus, our ability to detect long‐term changes in nest timing in many turtle populations may be constrained by sample size, speaks to the value of long‐term studies, and argues for the continuation of those currently in place.

Furthermore, among sea turtles, most of the nesting phenology research done to date has focused on only two species (with complicated migratory cycles), while most of the work done on nonmarine turtles has focused on *Chelydra* and *Chrysemys* (Table 1). Studies of other taxa, especially those in regions other than temperate North America, are sorely needed.

While our study provides insight into how morphological and environmental variability influences nesting dates of *Chelydra* and *Chrysemys* in Nebraska, these effects are complex and at times likely synergistic or antagonistic. Regardless, this and other studies demonstrate that turtles exhibit extensive plasticity in their reproductive biology. For example, from this study, the range of dates of first nesting varied across years by 19 days for *Chelydra* and 43 days for *Chrysemys*. However, it remains to be seen whether this plasticity is sufficient to overcome the threat of additional significant climate change, particularly warming (McGaugh et al., 2010; Refsnider & Janzen, 2016; Schwanz & Janzen, 2008; Urban et al., 2014).

## CONFLICT OF INTEREST

None declared.

## AUTHOR CONTRIBUTION

**Ashley R. Hedrick:** Conceptualization (equal); Investigation (equal); Writing‐original draft (equal); Writing‐review & editing (equal). **Daniel U. Greene:** Data curation (equal); Software (equal); Writing‐original draft (equal); Writing‐review & editing (equal). **Erin L. Lewis:** Data curation (equal); Investigation (equal); Writing‐original draft (equal); Writing‐review & editing (equal). **Andrew S. Hood:** Data curation (equal); Investigation (equal); Writing‐original draft (equal). **John B. Iverson:** Conceptualization (equal); Data curation (equal); Investigation (equal); Software (equal); Writing‐original draft (equal); Writing‐review & editing (equal).

## Supporting information

Tables S1‐S10

## Data Availability

The original phenology data files for *Chelydra* and *Chrysemys* are available from the DRYAD Digital Repository: https://doi.org/10.5061/dryad.2v6wwpzkn.

## References

[ece37105-bib-0001] Ahas, R. (1999). Long‐term phyto‐, ornitho‐ and ichthyophenological time‐series analyses in Estonia. International Journal of Biometeorology, 42, 119–123.

[ece37105-bib-0002] Avery, H. W. , Spotila, J. R. , Congdon, J. D. , Fischer, R. U. Jr , Standora, E. A. , & Avery, S. B. (1993). Roles of diet protein and temperature in the growth and nutritional energetics of juvenile Slider Turtles, *Trachemys scripta* . Physiological Zoology, 66, 902–925.

[ece37105-bib-0003] Bates, D. , Maechler, M. , Bolker, B. , & Walker, S. (2015). Fitting Linear Mixed‐Effects Models using lme4. Journal of Statistical Software, 67(1), 1–48.

[ece37105-bib-0004] Benard, M. F. (2015). Warming winters reduce frog fecundity and shift breeding phenology, which consequently alters larval development and metamorphic timing. Global Change Biology, 21, 1058–1065. 25263760 10.1111/gcb.12720

[ece37105-bib-0005] Bowers, K. E. , Grindstaff, J. L. , Soukup, S. S. , Drilling, N. E. , Eckerle, K. P. , Sakaluk, S. K. , & Thompson, C. F. (2016). Spring temperatures influence selection on breeding date and the potential for phenological mismatch in a migratory bird. Ecology, 97, 2880–2891. 10.1002/ecy.1516 27859132 PMC5119899

[ece37105-bib-0006] Bradey, N. L. , Leopold, A. C. , Ross, J. , & Huffaker, W. (1999). Phenological changes reflect climate change in Wisconsin. Proceedings of the National Academy of Sciences of the United States of America, 96, 9701–9704. 10.1073/pnas.96.17.9701 10449757 PMC22273

[ece37105-bib-0007] Buckardt, E. M. , Glowacki, G. A. , & Gibbs, J. P. (2020). Environmental cues that trigger nesting by Blanding’s Turtles (*Emydoidea blandingii*). Chelonian Conservation and Biology, 19, 67–71.

[ece37105-bib-0008] Bull, M. C. , & Burzacott, D. (2002). Changes in climate and in timing of pairing of the Australian lizard. *Tiliqua rugosa*: A 15 year study. Journal of Zoology, 256, 383–387.

[ece37105-bib-0009] Butler, C. J. (2019). A review of the effects of climate change on chelonians. Diversity, 11, 138 (22 pages). 10.3390/d11080138

[ece37105-bib-0010] Callard, I. P. , Lance, V. , Salhanick, A. R. , & Barad, D. (1978). The annual ovarian cycle of *Chrysemys picta*: Correlated changes in plasma steroids and parameters of vitellogenesis. General and Comparative Endocrinology, 35, 245–257. 10.1016/0016-6480(78)90069-2 689359

[ece37105-bib-0011] Carroll, D. M. , & Ultsch, G. R. (2007). Emergence season and survival in the nest of hatchling turtles in southcentral New Hampshire. Northeastern Naturalist, 14, 307–310.

[ece37105-bib-0012] Charmantier, A. , & Gienapp, P. (2014). Climate change and the timing of avain breeding and migration: Evolutionary versus plastic changes. Evolutionary Applications, 7, 15–28. 24454545 10.1111/eva.12126 PMC3894895

[ece37105-bib-0013] Christens, E. , & Bider, J. R. (1987). Nesting activity and hatchling success of the Painted Turtle (*Chrysemys picta marginata*) in southwestern Quebec. Herpetologica, 43, 55–65.

[ece37105-bib-0014] Congdon, J. D. , Breitenbach, G. L. , Van Loben Sels, R. C. , & Tinkle, D. W. (1987). Reproduction and nesting ecology of Snapping Turtles (*Chelydra serpentina*) in southeastern Michigan. Herpetologica, 43, 39–54.

[ece37105-bib-0015] Congdon, J. D. , Tinkle, D. W. , Breitenbach, G. L. , & van Loben Sels, R. C. (1983). Nesting ecology and hatching success in the turtle *Emydoidea blandingi* . Herpetologica, 39, 417–429.

[ece37105-bib-0016] Costanzo, J. P. , Iverson, J. B. , Wright, M. F. , & Lee, R. E. (1995). Cold hardiness and overwintering strategies of hatchlings in an assemblage of northern turtles. Ecology, 76, 1772–1785. 10.2307/1940709

[ece37105-bib-0017] Crick, H. Q. , Dudley, C. , Glue, D. E. , & Thomson, D. L. (1997). UK birds are laying eggs earlier. Nature, 388, 526 10.1038/41453

[ece37105-bib-0018] Czaja, R. A. , Kanonik, A. , & Burke, R. L. (2018). The effect of rainfall on predation of Diamond‐Backed Terrapin (*Malaclemys terrapin*) nests. Journal of Herpetology, 52, 402–405.

[ece37105-bib-0019] Dalleau, M. , Ciccione, S. , Mortimer, J. A. , Garnier, J. , Benhamou, S. , & Bourje, J. (2012). Nesting phenology of marine turtles: Insights from a regional comparative analysis on green turtle (*Chelonia mydas*). PLoS One, 7, e46920 10.1371/journal.pone.0046920 23056527 PMC3467270

[ece37105-bib-0020] Edge, C. B. , Rollinson, N. , Brooks, R. J. , Congdon, J. D. , Iverson, J. B. , Janzen, F. J. , & Litzgus, J. D. (2017). Phenotypic plasticity of nest timing in a post‐glacial landscape: How to reptiles adapt to seasonal time constraints? Ecology, 98, 512–524. 27870008 10.1002/ecy.1665

[ece37105-bib-0021] Ernst, C. H. , & Lovich, J. E. (2009). Turtles of the United States and Canada. Johns Hopkins University Press.

[ece37105-bib-0022] Fritts, S. R. , Grisham, B. A. , Cox, R. D. , Boal, C. W. , Haukos, D. A. , McDaniel, P. , Hagen, C. A. , & Greene, D. U. (2018). Interactive effects of severe drought and grazing on the life history cycle of a bioindicator species. Ecology and Evolution, 8, 9550–9562. 10.1002/ece3.4432 30377522 PMC6194261

[ece37105-bib-0023] Geller, G. A. (2012). Notes on the nesting ecology of Ouachita Map Turtles (*Graptemys ouachitensis*) at two Wisconsin sites using trail camera monitoring. Chelonian Conservation and Biology, 11, 206–213. 10.2744/CCB-0990.1

[ece37105-bib-0024] Gibbons, J. W. (1970). Reproductive dynamics of a turtle (*Pseudemys scripta*) population in a reservoir receiving heated effluent from a nuclear reactor. Canadian Journal of Zoology, 48, 881–885.

[ece37105-bib-0025] Gibbs, J. P. , & Breisch, A. R. (2001). Climate warming and calling phenology of frogs near Ithaca, New York, 1900–1999. Conservation Biology, 15, 1175–1178. 10.1046/j.1523-1739.2001.0150041175.x

[ece37105-bib-0026] Hawkes, L. A. , Broderick, A. C. , Godfrey, M. H. , & Godley, B. J. (2007). Investigating the potential impacts of climate change on a marine turtle population. Global Change Biology, 13, 923–932. 10.1111/j.1365-2486.2007.01320.x

[ece37105-bib-0027] Hays, G. C. , Broderick, A. C. , Godfrey, M. H. , & Godley, B. J. (2002). Climate change and sea turtles: A 150‐year reconstruction of incubation temperatures at a major marine rockery. Global Change Biology, 9, 642–646.

[ece37105-bib-0028] Hedrick, A. R. , Klondaris, H. M. , Corichi, L. C. , Dreslik, M. J. , & Iverson, J. B. (2018). The effects of climate on annual variation in reproductive output in Snapping Turtles (*Chelydra serpentina*). Canadian Journal of Zoology, 96, 221–228.

[ece37105-bib-0029] Holm, S. (1979). A simple sequentially rejective multiple test procedure. Scandinavian Journal of Statistics, 6, 65–70.

[ece37105-bib-0031] Iverson, J. B. (1991). Life history and demography of the Yellow Mud Turtle, *Kinosternon flavescens* . Herpetologica, 47, 371–393.

[ece37105-bib-0032] Iverson, J. B. , Griffiths, C. , Higgins, H. , & Sirulnik, A. G. (1997). Local and geographic variation in the reproductive biology of the Snapping Turtle (*Chelydra serpentina*). Herpetologica, 53, 96–117.

[ece37105-bib-0033] Iverson, J. B. , & Smith, G. R. (1993). Reproductive ecology of the Painted Turtle (*Chrysemys picta*) in the Nebraska Sandhills. Copeia, 1, 1–21.

[ece37105-bib-0034] Janzen, F. J. , Hoekstra, L. A. , Brooks, R. J. , Carroll, D. M. , Gibbons, J. W. , Greene, J. L. , Iverson, J. B. , Litzgus, J. D. , Michael, E. D. , Parren, S. G. , Roosenburg, W. M. , Strain, G. F. , Tucker, J. K. , & Ultsch, G. R. (2018). Altered spring phenology of North American freshwater turtles and the importance of representative populations. Ecology and Evolution, 8, 5815–5827. 10.1002/ece3.4120 29938095 PMC6010881

[ece37105-bib-0035] Janzen, F. J. , & Paukstis, G. L. (1991). Environmental sex determination in reptiles: Ecology, evolution, and experimental design. Quarterly Review of Biology, 66, 149–179. 10.1086/417143 1891591

[ece37105-bib-0036] Jara, F. G. , Thurman, L. L. , Montiglio, P.‐O. , Sih, A. , & Garcia, T. S. (2019). Warming‐induced shifts in amphibian phenology and behavior lead to altered predator‐prey dynamics. Oecologia, 189, 803–813. 10.1007/s00442-019-04360-w 30810801

[ece37105-bib-0037] Kosaka, Y. , & Xie, S.‐P. (2013). Recent global‐warming hiatus tied to equatorial Pacific surface cooling. Nature, 501, 403–407. 10.1038/nature12534 23995690

[ece37105-bib-0038] Lamont, M. M. , & Fujisaki, I. (2014). Effects of ocean temperature on nesting phenology and fecundity of the Loggerhead Sea Turtle (*Caretta caretta*). Journal of Herpetology, 48, 98–102.

[ece37105-bib-0039] Levengood, J. , Hanley, K. , & Rostal, D. (2015). Annual variation and timing of nesting in *Gopherus polyphemus* from 1994 to 2014 In 13th Annual Symposium on the Conservation and Biology of Tortoises and Freshwater Turtles. Tucson, Arizona.

[ece37105-bib-0040] Loarie, S. R. , Duffy, P. B. , Hamilton, H. , Asner, G. P. , Field, C. B. , & Ackerly, D. D. (2009). The velocity of climate change. Nature, 462, 1052–1055. 10.1038/nature08649 20033047

[ece37105-bib-0041] Lovich, J. E. , Agha, M. , Meulblok, M. , Meyer, K. , Ennen, J. , Loughran, C. , Madrak, S. , & Bjurlin, C. (2012). Climatic variation affects clutch phenology in Agassiz’s Desert Tortoise *Gopherus agassizii* . Endangered Species Research, 19, 63–74. 10.3354/esr00463

[ece37105-bib-0042] Lüdecke, D. (2018). ggeffects: Tidy data frames of marginal effects from regression models. Journal of Open Source Software, 3(26), 772 10.21105/joss.00772

[ece37105-bib-0043] Mazaris, A. D. , Kallimanis, A. S. , Pantis, J. D. , & Hays, G. C. (2012). Phenological response of sea turtles to environmental variation across a species' northern range. Proceedings of the Royal Society B: Biological Sciences, 280, 20122397. 10.1098/rspb.2012.2397 PMC3574410 23193130

[ece37105-bib-0044] Mazaris, A. D. , Kallimanis, A. S. , Tzanopoulos, J. , Sgardelis, S. P. , & Pantis, J. D. (2008). Do long‐term changes in sea surface temperature at breeding areas affect the breeding dates and reproduction performance of Mediterranean Loggerhead Turtles? Implications for climate change. Journal of Experimental Marine Biology and Ecology, 367, 219–226.

[ece37105-bib-0045] Mazaris, A. D. , Kallimanis, A. S. , Tzanopoulos, J. , Sgardelis, S. P. , & Pantis, J. D. (2009). Sea surface temperature variations in core foraging grounds drive nesting trends and phenology of Loggerhead Turtles in the Mediterranean Sea. Journal of Experimental Marine Biology and Ecology, 379, 23–27. 10.1016/j.jembe.2009.07.026

[ece37105-bib-0046] McGaugh, S. E. , Schwanz, L. E. , Bowden, R. M. , Gonzalez, J. E. , & Janzen, F. J. (2010). Inheritance of nesting behavior across natural environmental variation in a turtle with temperature‐dependent sex determination. Proceedings of the Royal Society B: Biological Science, 277, 1219–1226. 10.1098/rspb.2009.1883 PMC2842811 20018783

[ece37105-bib-0047] Mitchell, T. S. , Refsnider, J. M. , Sethuraman, A. , Warner, D. A. , & Janzen, F. J. (2017). Experimental assessment of winter conditions on turtle nesting behavior. Evolutionary Ecology Research, 18, 271–280.

[ece37105-bib-0048] Morenro‐Rueda, G. , Pleguezuelos, J. M. , & Alaminos, E. (2009). Climate warming and activity period extensions in the Mediterranean snake *Malpolon monspessulanus* . Climate Change, 92, 235–242.

[ece37105-bib-0049] Muir, T. J. , Dishong, B. D. , Costanzo, J. P. , & Lee, R. E. (2012). Energy use in terrestrially hibernating hatchling turtles (*Chrysemys picta*) is extremely sensitive to overwintering temperature. Integrative and Comparative Biology, 52, e300.

[ece37105-bib-0050] Nagle, R. , & Congdon, J. D. (2016). Reproductive ecology of *Graptemys geographica* of the Juniata River in central Pennsylvania, with recommendations for conservation. Herpetological Conservation and Biology, 11, 232–243.

[ece37105-bib-0051] Neeman, N. , Robinson, N. J. , Paladino, F. V. , Spotila, J. R. , & O’Connor, M. P. (2015). Phenology shifts in Leatherback Turtles (*Dermochelys coriacea*) due to change in sea surface temperatures. Journal of Experimental Marine Biology and Ecology, 462, 113–120.

[ece37105-bib-0052] Obbard, M. E. , & Brooks, R. J. (1987). Prediction of the onset of annual nesting season of the Common Snapping Turtle, *Chelydra serpentina* . Herpetologica, 43, 324–328.

[ece37105-bib-0053] Pachauri, R. K. , Allen, M. R. , Barros, V. R. , Broome, J. , Cramer, W. , Christ, R. , Church, J. A. , Clarke, L. , Dahe, Q. , Dasgupta, P. , Dubash, N. K. , Edenhofer, O. , Elgizouli, I. , Field, C. B. , Forster, P. , Friedlingstein, P. , Fuglestvedt, J. , Gomez‐Echeverri, L. , Hallegatte, S. , … van Ypserle, J. P. (2014). Climate Change 2014: Synthesis Report In R. Pachauri , & L. Meyer (Eds.), Contribution of Working Groups I, II and III to the Fifth Assessment Report of the Intergovernmental Panel on Climate Change (151 pp). IPCC.

[ece37105-bib-0054] Parmesan, C. , & Yohe, G. (2003). A globally coherent fingerprint of climate change impacts across natural systems. Nature, 421, 37–42. 10.1038/nature01286 12511946

[ece37105-bib-0055] Patel, S. H. , Morreale, S. J. , Saba, V. S. , Panagopoulou, A. , Margaritoulis, D. , & Spotila, J. R. (2016). Climate impacts on sea turtle breeding phenology in Greece and associated foraging habitats in the wider Mediterranean region. PLoS One, 11(6), e0157170 10.1371/journal.pone.0157170 27332550 PMC4917093

[ece37105-bib-0056] Pike, D. A. (2008). Environmental correlates of nesting in Loggerhead Turtles *Caretta caretta* . Animal Behavior, 76, 603–610. 10.1016/j.anbehav.2008.04.010

[ece37105-bib-0057] Pike, D. A. (2009). Do green turtles modify their nesting seasons in response to environmental temperatures? Chelonian Conservation and Biology, 8, 43–47. 10.2744/CCB-0726.1

[ece37105-bib-0058] Pike, D. A. , Antworth, R. L. , & Stiner, J. C. (2006). Earlier nesting contributes to shorter nesting seasons for the Loggerhead Sea Turtle, *Caretta caretta* . Journal of Herpetology, 40, 91–94. 10.1670/100-05N.1

[ece37105-bib-0059] Price, M. , & Waser, N. (1998). Effects of experimental warming on plants. Ecology, 79, 1261–1271.

[ece37105-bib-0060] R Core Team (2017). R: A language and environment for statistical computing. R Foundation for Statistical Computing https://www.R‐project.org/

[ece37105-bib-0061] Réale, D. , McAdam, A. G. , Boutin, S. , & Berteaux, D. (2003). Genetic and plastic responses of a northern mammal to climate change. Proceedings of the Royal Society of London. Series B: Biological Sciences, 270, 591–596. 10.1098/rspb.2002.2224 12769458 PMC1691280

[ece37105-bib-0062] Refsnider, J. M. , & Janzen, F. J. (2016). Temperature‐dependent sex determination under rapid anthropogenic environmental change: Evolution at a turtle’s pace. Journal of Heredity, 2016, 61–70. 10.1093/jhered/esv053 26245920

[ece37105-bib-0063] Rhen, T. , & Lang, J. W. (1999). Temperature during embryonic and juvenile development influences growth in hatchling Snapping Turtles, *Chelydra serpentina* . Journal of Thermal Biology, 24, 33–41. 10.1016/S0306-4565(98)00035-7

[ece37105-bib-0064] Rollinson, N. , Farmer, R. G. , & Brooks, R. J. (2012). Widespread reproductive variation in North American turtles: Temperature, egg size and optimality. Zoology, 115, 160–169. 10.1016/j.zool.2011.10.005 22541670

[ece37105-bib-0065] Root, T. L. , Price, J. T. , Hall, K. R. , Schneider, S. H. , Rosenzweig, C. , & Pounds, J. (2003). Fingerprints of global warming on wild animals and plants. Nature, 421, 57–60. 10.1038/nature01333 12511952

[ece37105-bib-0066] Rowe, J. W. , Coval, K. A. , & Campbell, K. C. (2003). Reproductive characteristics of female Midland Painted Turtles (*Chrysemys picta marginata*) from a population on Beaver Island, Michigan. Copeia, 2003, 326–336.

[ece37105-bib-0067] Roy, D. B. , & Sparks, T. H. (2000). Phenology of British butterflies and climate change. Global Change Biology, 6, 407–416. 10.1046/j.1365-2486.2000.00322.x

[ece37105-bib-0068] Saino, N. , Ambrosini, R. , Rubolini, D. , von Hardenberg, J. , Provenzale, A. , Hüppop, K. , Hüppop, O. , Lehikoinen, A. , Lehikoinen, E. , Rainio, K. , Romano, M. , & Sokolov, L. (2011). Climate warming, ecological mismatch at arrival and population decline in migratory birds. Proceedings of the Royal Society of London. Series B: Biological Sciences, 278, 835–842. 10.1098/rspb.2010.1778 20861045 PMC3049050

[ece37105-bib-0069] Schofield, G. , Bishop, C. M. , Katselidis, K. A. , Dimopoulos, P. , Pantis, J. D. , & Hays, G. C. (2009). Microhabitat selection by sea turtles in a dynamic thermal marine environment. Journal of Animal Ecology, 78, 14–21. 10.1111/j.1365-2656.2008.01454.x 18699794

[ece37105-bib-0070] Schwanz, L. E. , & Janzen, F. J. (2008). Climate change and temperature‐dependent sex determination: Can individual plasticity in nesting phenology prevent extreme sex ratios? Physiological and Biochemical Zoology, 81, 826–834. 10.1086/590220 18831689

[ece37105-bib-0071] Sherry, R. A. , Zhou, X. , Gu, S. , Arnone, J. A. , Schimel, D. S. , Verburg, P. S. , Wallace, L. L. , & Luo, Y. (2007). Divergence of reproductive phenology under climate warming. Proceedings of the National Academy of Sciences of the United States of America, 104, 198–202. 10.1073/pnas.0605642104 17182748 PMC1713188

[ece37105-bib-0072] Stanford, C. B. , Iverson, J. B. , Rhodin, A. G. J. , van Dijk, P. P. , Mittermeier, R. A. , Kuchling, G. , Berry, K. H. , Bertolero, A. , Bjorndal, K. A. , Blanck, T. E. G. , Buhlmann, K. A. , Burke, R. L. , Congdon, J. C. , Diagne, T. , Edwards, T. , Eisemberg, C. C. , Ennen, J. R. , Forero‐Medina, G. , Frankel, M. , … Walde, A. W. (2020). Turtles in trouble. Current Biology, 30, R721–R735. 32574638 10.1016/j.cub.2020.04.088

[ece37105-bib-0073] Telemeco, R. S. , Elphick, M. J. , & Shine, R. (2009). Nesting lizards (*Brassiana duperreyi*) compensate partly, but not completely for climate change. Ecology, 90, 17–22. 19294908 10.1890/08-1452.1

[ece37105-bib-0074] Tucker, J. K. , Dolan, C. R. , Lamer, J. T. , & Dustman, E. A. (2008). Climatic warming, sex ratios and red‐eared slider (*Trachemys scripta elegans*) in Illinois. Chelonian Conservation and Biology, 7, 60–69.

[ece37105-bib-0075] Urban, M. C. , Richardson, J. L. , & Friedenfelds, N. A. (2014). Plasticity and the genetic adaptation mediate amphibian and reptile responses to climate change. Evolutionary Applications, 7, 88–103. 24454550 10.1111/eva.12114 PMC3894900

[ece37105-bib-0076] Weishampel, J. F. , Bagley, D. A. , & Ehrhart, L. M. (2004). Earlier nesting by Loggerhead Sea Turtles following sea surface warming. Global Change Biology, 10, 1424–1427. 10.1111/j.1529-8817.2003.00817.x

[ece37105-bib-0077] Weishampel, J. F. , Bagley, D. A. , Ehrhart, L. M. , & Weishampel, A. C. (2010). Nesting phenologies of two sympatric sea turtle species related to sea surface temperatures. Endangered Species Research, 12, 41–47. 10.3354/esr00290

[ece37105-bib-0078] While, G. M. , & Uller, T. (2014). Quo vadis amphibian? Global warming and breeding phenology in frogs, toads, and salamanders. Ecography, 37, 921–929.

[ece37105-bib-0079] Wood, R. , Herlands, R. , Baker, P. , Boerner, R. , & Atkinson, B. (2013). Carnage on the causeway: twenty‐two years of Diamondback Terrapin (*Malaclemys terrapin*) road kills on southern New Jersey coastal roads In 11th Annual Symposium on the Conservation and Biology of Tortoises and Freshwater Turtles. St. Louis, Missouri.

