# Supplementary material for: Climate effects on nesting phenology in Nebraska turtles
Source: Ecol Evol. 2021 Jan 8;11(3):1225–39. doi: 10.1002/ece3.7105 (PMC7863389; doi:10.1002/ece3.7105)
Supplement: Supplementary file 1 — Tables S1‐S10 [file ECE3-11-1225-s001.docx]

**SUPPLEMENTAL MATERIAL**

**Supplemental Table 1** Candidate models for an analysis to evaluate differences between body size metrics between Gimlet and Island Lakes (response variable) for *Chelydra serpentina* from 1993 through 2017 at Crescent Lake National Wildlife Refuge, Garden County, Nebraska.

| **Model** | **K** | **AIC** | **ΔAIC** | **Wt** | **Cum.Wt** |
| --- | --- | --- | --- | --- | --- |
| Clutch size | 2 | 236.94 | 0.00 | 1.00 | 1.00 |
| Null | 1 | 279.01 | 42.07 | 0.00 | 1.00 |
| Carapace length | 2 | 280.95 | 44.01 | 0.00 | 1.00 |

**Supplemental Table 2** Model results for an analysis to evaluate differences between body size metrics between Gimlet and Island Lakes (response variable) for *Chelydra serpentina* from 1993 through 2017 at Crescent Lake National Wildlife Refuge, Garden County, Nebraska. Asterisks indicate variable significance.

| **Variable** | **Estimate** | **SE** | **LCI** | **UCI** |  |
| --- | --- | --- | --- | --- | --- |
| Intercept | -1.57 | 0.19 | -1.94 | -1.20 |  |
| Clutch size | -1.16 | 0.20 | -1.56 | -0.76 | * |
| Carapace length | -0.04 | 0.15 | -0.33 | 0.26 |  |

**Supplemental Table 3** Candidate models for an analysis to evaluate changes in body size metrics over time (year = response variable) for *Chelydra serpentina* (1993 through 2017) and *Chrysemys picta* (1986 through 2017) at Crescent Lake National Wildlife Refuge, Garden County, Nebraska.

| **Analysis** | **Model** | **K** | **AIC** | **ΔAIC** | **Wt** | **Cum.Wt** |
| --- | --- | --- | --- | --- | --- | --- |
| *Chelydra* | Carapace length | 4 | 1698.2 | 0.0 | 0.34 | 0.34 |
|  | Null | 3 | 1698.4 | 0.2 | 0.31 | 0.65 |
|  | Clutch size | 4 | 1699.2 | 1.0 | 0.20 | 0.85 |
|  | Mean egg mass | 4 | 1699.7 | 1.5 | 0.15 | 1.00 |
| *Chrysemys* | Carapace length | 4 | 2311.5 | 0.0 | 0.96 | 0.96 |
|  | Null | 3 | 2318.5 | 7.0 | 0.03 | 0.99 |
|  | Clutch size | 4 | 2320.4 | 8.9 | 0.01 | 1.00 |

**Supplemental Table 4** Model results from analyses to evaluate changes in body size metrics over time (year = response variable) during our study of *Chelydra serpentina* (1993 through 2017) and *Chrysemys picta* (1986 through 2017) at Crescent Lake National Wildlife Refuge, Garden County, Nebraska. Asterisks indicate variable significance.

| **Analysis** | **Variable** | **Estimate** | **SE** | **LCI** | **UCI** |  |
| --- | --- | --- | --- | --- | --- | --- |
| *Chelydra* | Intercept | 2008 | 1.61 | 2005 | 2011 |  |
|  | Carapace length | -0.56 | 0.37 | -1.29 | 0.18 |  |
|  | Clutch size | -0.44 | 0.40 | -1.24 | 0.35 |  |
|  | Mean egg mass | -0.31 | 0.38 | -1.05 | 0.44 |  |
| *Chrysemys* | Intercept | 2006 | 0.73 | 2004 | 2007 |  |
|  | Carapace length | 1.11 | 0.31 | 0.46 | 1.77 | * |
|  | Clutch size | 0.02 | 0.10 | -0.18 | 0.23 |  |

**Supplemental Table 5** Candidate models from an analysis of the effects of climatic variables on Julian day of nest deposition (response variable) for *Chelydra serpentina* from 1993 through 2017 at Crescent Lake National Wildlife Refuge, Garden County, Nebraska.

| **Model** | **K** | **AICc** | **ΔAICc** | **Wt** | **Cum.Wt** |
| --- | --- | --- | --- | --- | --- |
| May minimum | 5 | 1715.3 | 0.0 | 0.54 | 0.54 |
| May maximum | 5 | 1717.5 | 2.2 | 0.18 | 0.72 |
| December maximum (preceding) | 5 | 1718.8 | 3.5 | 0.09 | 0.81 |
| July rain (preceding) | 5 | 1719.6 | 4.3 | 0.06 | 0.87 |
| April maximum | 5 | 1721.7 | 6.4 | 0.02 | 0.89 |
| December minimum (preceding) | 5 | 1723.1 | 7.8 | 0.01 | 0.90 |
| January rain (preceding) | 5 | 1723.2 | 7.9 | 0.01 | 0.91 |
| January maximum | 5 | 1723.2 | 7.9 | 0.01 | 0.92 |
| March maximum | 5 | 1723.6 | 8.3 | 0.01 | 0.93 |
| April minimum | 5 | 1723.8 | 8.5 | 0.01 | 0.94 |
| Null | 4 | 1725.0 | 9.7 | 0.00 | 0.94 |
| February rain | 5 | 1725.3 | 10.0 | 0.00 | 0.95 |
| August rain (preceding) | 5 | 1725.3 | 10.0 | 0.00 | 0.95 |
| March minimum | 5 | 1725.3 | 10.0 | 0.00 | 0.96 |
| May rain | 5 | 1725.6 | 10.3 | 0.00 | 0.96 |
| April rain | 5 | 1725.7 | 10.4 | 0.00 | 0.96 |
| December rain (preceding) | 5 | 1725.7 | 10.4 | 0.00 | 0.96 |
| September maximum (preceding) | 5 | 1725.8 | 10.5 | 0.00 | 0.97 |
| March rain | 5 | 1725.8 | 10.5 | 0.00 | 0.97 |
| September rain (preceding) | 5 | 1726.0 | 10.7 | 0.00 | 0.97 |
| July minimum (preceding) | 5 | 1726.2 | 10.9 | 0.00 | 0.97 |
| September minimum (preceding) | 5 | 1726.2 | 10.9 | 0.00 | 0.98 |
| October rain (preceding) | 5 | 1726.2 | 10.9 | 0.00 | 0.98 |
| July maximum (preceding) | 5 | 1726.4 | 11.1 | 0.00 | 0.98 |
| February maximum | 5 | 1726.4 | 11.1 | 0.00 | 0.98 |
| January minimum | 5 | 1726.5 | 11.2 | 0.00 | 0.99 |
| August minimum (preceding) | 5 | 1726.5 | 11.2 | 0.00 | 0.99 |
| October maximum (preceding) | 5 | 1726.5 | 11.2 | 0.00 | 0.99 |
| November maximum (preceding) | 5 | 1726.5 | 11.2 | 0.00 | 0.99 |
| February minimum | 5 | 1726.7 | 11.4 | 0.00 | 0.99 |
| November rain (preceding) | 5 | 1726.7 | 11.4 | 0.00 | 1.00 |
| August maximum (preceding) | 5 | 1726.9 | 11.6 | 0.00 | 1.00 |
| November minimum (preceding) | 5 | 1727.0 | 11.7 | 0.00 | 1.00 |
| October minimum (preceding) | 5 | 1727.0 | 11.7 | 0.00 | 1.00 |
|  |  |  |  |  |  |

**Supplemental Table 6** Model results from an analysis of the effects of climatic variables on Julian day of nest deposition (response variable) for *Chelydra serpentina* from 1993 through 2017 at Crescent Lake National Wildlife Refuge, Garden County, Nebraska. Asterisks indicate variable significance.

| **Variable** | **Estimate** | **SE** | **LCI** | **UCI** |  |
| --- | --- | --- | --- | --- | --- |
| (Intercept) | 162.76 | 1.02 | 160.75 | 164.77 |  |
| May minimum | -3.70 | 0.89 | -5.46 | -1.94 | * |
| May maximum | -3.41 | 0.96 | -5.29 | -1.53 | * |
| December maximum (preceding) | -3.61 | 1.10 | -5.78 | -1.44 | * |
| July rain (preceding) | 3.31 | 1.09 | 1.18 | 5.45 | * |
| April maximum | -2.90 | 1.16 | -5.17 | -0.62 | * |
| December minimum (preceding) | -2.48 | 1.18 | -4.80 | -0.16 | * |
| January rain (preceding) | -2.34 | 1.12 | -4.55 | -0.13 | * |
| January maximum | -2.39 | 1.17 | -4.68 | -0.10 | * |
| March maximum | -2.24 | 1.15 | -4.50 | 0.02 |  |
| April minimum | -2.29 | 1.22 | -4.69 | 0.11 |  |
| February rain | 1.64 | 1.21 | -0.75 | 4.03 |  |
| August rain (preceding) | 1.72 | 1.27 | -0.79 | 4.22 |  |
| March minimum | -1.78 | 1.33 | -4.39 | 0.83 |  |
| May rain | 1.42 | 1.19 | -0.92 | 3.77 |  |
| April rain | 1.45 | 1.27 | -1.05 | 3.96 |  |
| December rain (preceding) | 1.54 | 1.35 | -1.11 | 4.19 |  |
| September maximum (preceding) | 1.64 | 1.49 | -1.29 | 4.58 |  |
| March rain | 1.57 | 1.44 | -1.27 | 4.41 |  |
| September rain (preceding) | -1.33 | 1.34 | -3.98 | 1.31 |  |
| July minimum (preceding) | -1.20 | 1.31 | -3.77 | 1.37 |  |
| September minimum (preceding) | 1.17 | 1.29 | -1.37 | 3.71 |  |
| October rain (preceding) | 1.54 | 1.72 | -1.85 | 4.93 |  |
| July maximum (preceding) | -1.00 | 1.26 | -3.48 | 1.49 |  |
| February maximum | -1.14 | 1.45 | -3.98 | 1.71 |  |
| January minimum | -0.87 | 1.21 | -3.26 | 1.51 |  |
| August minimum (preceding) | -0.88 | 1.24 | -3.32 | 1.57 |  |
| October maximum (preceding) | -0.91 | 1.30 | -3.47 | 1.65 |  |
| November maximum (preceding) | -0.83 | 1.21 | -3.20 | 1.55 |  |
| February minimum | -0.70 | 1.21 | -3.08 | 1.67 |  |
| November rain (preceding) | -0.82 | 1.50 | -3.78 | 2.14 |  |
| August maximum (preceding) | -0.43 | 1.24 | -2.86 | 2.01 |  |
| November minimum (preceding) | 0.23 | 1.30 | -2.33 | 2.79 |  |
| October minimum (preceding) | 0.14 | 1.26 | -2.34 | 2.63 |  |

**Supplemental Table 7** Model results from an analysis to evaluate differences between reproductive metrics for *Chrysemys picta* and predicted Julian day of nest deposition from 1986 through 2017 at Crescent Lake National Wildlife Refuge, Garden County, Nebraska.

| **Predictor** | **K** | **AIC** | **ΔAIC** | **Wt** | **Cum.Wt** |  |
| --- | --- | --- | --- | --- | --- | --- |
| February minimum | 6 | 3349.8 | 0.0 | 0.15 | 0.15 |  |
| September minimum (preceding) | 6 | 3350.0 | 0.2 | 0.13 | 0.29 |  |
| October minimum (preceding) | 6 | 3350.4 | 0.6 | 0.11 | 0.40 |  |
| April rain | 6 | 3351.3 | 1.5 | 0.07 | 0.47 |  |
| December minimum (preceding) | 6 | 3351.5 | 1.7 | 0.07 | 0.53 |  |
| February maximum | 6 | 3351.7 | 1.9 | 0.06 | 0.59 |  |
| Null | 5 | 3353.1 | 3.3 | 0.03 | 0.62 |  |
| April maximum | 6 | 3352.2 | 2.4 | 0.03 | 0.65 |  |
| December rain (preceding) | 6 | 3353.3 | 3.5 | 0.03 | 0.67 |  |
| May maximum | 6 | 3353.6 | 3.8 | 0.02 | 0.70 |  |
| January minimum | 6 | 3353.9 | 4.1 | 0.02 | 0.72 |  |
| March rain | 6 | 3354.0 | 4.2 | 0.02 | 0.73 |  |
| October rain (preceding) | 6 | 3354.3 | 4.5 | 0.02 | 0.75 |  |
| January rain | 6 | 3354.4 | 4.6 | 0.02 | 0.77 |  |
| September maximum (preceding) | 6 | 3354.4 | 4.6 | 0.02 | 0.78 |  |
| February rain | 6 | 3354.7 | 4.9 | 0.01 | 0.79 |  |
| September rain (preceding) | 6 | 3354.7 | 4.9 | 0.01 | 0.81 |  |
| October maximum (preceding) | 6 | 3354.7 | 4.9 | 0.01 | 0.82 |  |
| December maximum (preceding) | 6 | 3354.8 | 5.0 | 0.01 | 0.83 |  |
| November minimum (preceding) | 6 | 3354.8 | 5.0 | 0.01 | 0.84 |  |
| January maximum | 6 | 3354.9 | 5.1 | 0.01 | 0.86 |  |
| July maximum (preceding) | 6 | 3355.0 | 5.2 | 0.01 | 0.87 |  |
| November maximum (preceding) | 6 | 3355.0 | 5.2 | 0.01 | 0.88 |  |
| July rain (preceding) | 6 | 3355.0 | 5.2 | 0.01 | 0.89 |  |
| May minimum | 6 | 3355.0 | 5.2 | 0.01 | 0.90 |  |
| May rain | 6 | 3355.0 | 5.2 | 0.01 | 0.91 |  |
| March maximum | 6 | 3355.0 | 5.2 | 0.01 | 0.92 |  |
| April minimum | 6 | 3355.0 | 5.2 | 0.01 | 0.93 |  |
| November rain (preceding) | 6 | 3355.0 | 5.2 | 0.01 | 0.95 |  |
| August minimum (preceding) | 6 | 3355.0 | 5.2 | 0.01 | 0.96 |  |
| July minimum (preceding) | 6 | 3355.1 | 5.3 | 0.01 | 0.97 |  |
| August rain (preceding) | 6 | 3355.1 | 5.3 | 0.01 | 0.98 |  |
| August maximum (preceding) | 6 | 3355.1 | 5.3 | 0.01 | 0.99 |  |
| March minimum | 6 | 3355.1 | 5.3 | 0.01 | 1.00 |  |

**Supplemental Table 8** Model results from an analysis to evaluate differences between reproductive metrics for *Chrysemys picta* and predicted Julian day of nest deposition from 1986 through 2017 at Crescent Lake National Wildlife Refuge, Garden County, Nebraska. Asterisks indicate variable significance.

| **Variable** | **Estimate** | **SE** | **LCI** | **UCI** |  |
| --- | --- | --- | --- | --- | --- |
| (Intercept) | 159.85 | 1.49 | 156.92 | 162.78 |  |
| February minimum | 3.01 | 1.22 | 0.61 | 5.41 | * |
| September minimum (preceding) | 2.93 | 1.22 | 0.54 | 5.32 | * |
| October minimum (preceding) | 2.76 | 1.21 | 0.39 | 5.13 | * |
| April rain | 3.05 | 1.50 | 0.10 | 6.00 | * |
| December minimum (preceding) | 2.47 | 1.24 | 0.04 | 4.90 | * |
| February maximum | 2.68 | 1.40 | -0.07 | 5.43 |  |
| April maximum | -2.29 | 1.64 | -5.51 | 0.94 |  |
| December rain (preceding) | 2.09 | 1.53 | -0.91 | 5.09 |  |
| May maximum | -2.03 | 1.63 | -5.23 | 1.16 |  |
| January minimum | 1.40 | 1.24 | -1.04 | 3.85 |  |
| March rain | -1.66 | 1.55 | -4.70 | 1.38 |  |
| October rain (preceding) | 1.37 | 1.48 | -1.54 | 4.27 |  |
| January rain | -1.15 | 1.33 | -3.76 | 1.47 |  |
| September maximum (preceding) | 1.29 | 1.52 | -1.69 | 4.27 |  |
| February rain | -0.80 | 1.29 | -3.33 | 1.73 |  |
| September rain (preceding) | 0.99 | 1.64 | -2.23 | 4.20 |  |
| October maximum (preceding) | 0.90 | 1.50 | -2.04 | 3.85 |  |
| December maximum (preceding) | 0.84 | 1.44 | -1.99 | 3.67 |  |
| November minimum (preceding) | 0.68 | 1.38 | -2.04 | 3.40 |  |
| January maximum | -0.55 | 1.35 | -3.21 | 2.11 |  |
| July maximum (preceding) | 0.50 | 1.40 | -2.26 | 3.25 |  |
| November maximum (preceding) | 0.47 | 1.39 | -2.26 | 3.20 |  |
| July rain (preceding) | 0.47 | 1.45 | -2.38 | 3.32 |  |
| May minimum | -0.46 | 1.47 | -3.33 | 2.42 |  |
| May rain | 0.47 | 1.70 | -2.87 | 3.80 |  |
| March maximum | -0.37 | 1.56 | -3.43 | 2.69 |  |
| April minimum | -0.33 | 1.57 | -3.42 | 2.76 |  |
| November rain (preceding) | 0.30 | 1.43 | -2.51 | 3.10 |  |
| August minimum (preceding) | -0.27 | 1.36 | -2.93 | 2.39 |  |
| July minimum (preceding) | 0.18 | 1.34 | -2.45 | 2.82 |  |
| August rain (preceding) | -0.15 | 1.37 | -2.84 | 2.54 |  |
| August maximum (preceding) | -0.07 | 1.44 | -2.90 | 2.75 |  |
| March minimum | -0.06 | 1.54 | -3.09 | 2.97 |  |

**Supplemental Table 9** Candidate models from analyses to evaluate differences between reproductive metrics and Julian day of nest deposition (response variable) for *Chelydra serpentina* (1993 through 2017) and *Chrysemys picta* (1986 through 2017) at Crescent Lake National Wildlife Refuge, Garden County, Nebraska.

| **Analysis** | **Model** | **K** | **AIC** | **ΔAIC** | **Wt** | **Cum.Wt** |
| --- | --- | --- | --- | --- | --- | --- |
| *Chelydra* | Carapace length | 5 | 1426.5 | 0.0 | 0.69 | 0.69 |
|  | Mean egg mass | 5 | 1428.4 | 1.9 | 0.26 | 0.95 |
|  | Clutch size | 5 | 1431.6 | 5.1 | 0.05 | 1.00 |
|  | Null | 4 | 1440.4 | 13.9 | 0.00 | 1.00 |
| *Chrysemys* | Carapace length | 5 | 2063.6 | 0.0 | 0.81 | 0.81 |
|  | Clutch size | 5 | 2067.1 | 3.5 | 0.14 | 0.95 |
|  | Null | 4 | 2069.0 | 5.4 | 0.05 | 1.00 |

**Supplemental Table 10** Model results from analyses to evaluate reproductive metrics and predicted nesting dates (response variable) for *Chelydra serpentina* (1993 through 2017) and *Chrysemys picta* (1986 through 2017) at Crescent Lake National Wildlife Refuge, Garden County, Nebraska.

| **Analysis** | **Variable** | **Estimate** | **SE** | **LCI** | **UCI** |  |
| --- | --- | --- | --- | --- | --- | --- |
| *Chelydra* | Intercept | 164.22 | 1.37 | 161.52 | 166.91 |  |
|  | Carapace length | -0.86 | 0.21 | -1.28 | -0.44 | * |
|  | Mean egg mass | -0.86 | 0.22 | -1.30 | -0.41 | * |
|  | Clutch size | -0.77 | 0.22 | -1.19 | -0.34 | * |
| *Chrysemys* | Intercept | 159.59 | 1.75 | 156.16 | 163.02 |  |
|  | Carapace length | -0.69 | 0.25 | -1.18 | -0.20 | * |
|  | Clutch size | -0.17 | 0.08 | -0.33 | -0.002 | * |

* indicates variable significance
